# Changes in the Relationship between Ionized and Total Calcium in Clinically Healthy Dairy Cows in the Period around Calving

**DOI:** 10.3390/ani11041036

**Published:** 2021-04-06

**Authors:** Dorothee Ott, Katharina T. Schrapers, Jörg R. Aschenbach

**Affiliations:** 1 Institute of Veterinary Physiology, Faculty of Veterinary Medicine, Freie Universität Berlin, 14163 Berlin, Germany; dorothee_ott@web.de; 2 PerformaNat GmbH, 14163 Berlin, Germany; schrapers@performanat.de

**Keywords:** dairy cows, hypocalcemia, ionized calcium, total calcium, heparin

## Abstract

**Simple Summary:**

Hypocalcemia is a widespread problem in dairy cows in the first days after calving, which increases the risk for secondary diseases. In practice, the measurement of total blood serum or plasma calcium is widely used to diagnose hypocalcemia. The present study demonstrates a high discrepancy between total and ionized calcium specifically around calving, suggesting that only ionized calcium provides an accurate indication of the animal’s calcium status during that period. We developed an optimized model for prediction of ionized calcium from total calcium, non-esterified fatty acids, beta-hydroxybutyric acid, cholesterol, and phosphorous. However, the precision of that model is still unsatisfactory.

**Abstract:**

We aimed to establish a model for prediction of iCa from tCa, using multivariable regressions with diverse blood constituents. Blood was taken from 14 cows at days −2, 0, 2, 4, 7, and 14 relative to parturition. Cows were clinically healthy, and no hypocalcaemia prophylaxis and treatment were applied. Total calcium and further parameters were determined from frozen serum. Ionized calcium, blood gases, and electrolytes were determined from heparin-stabilized blood samples. Linear regression between iCa and tCa was estimated. Precision improved only slightly using a multivariable model. Best precision was achieved when estimating the iCa:tCa ratio from other blood constituents. To identify the reason behind the poorly predictive value of tCa for iCa, the relative changes of iCa and tCa around calving were calibrated to the respective values of day −2 (=100%) for each cow. An increase in the iCa:tCa ratio was observed from 0.43 at day −2 to 0.48 at day 0, followed by a gradual decrease towards 0.43 at day 7. We conclude that routine measurement of iCa should be implemented in the diagnosis of hypocalcaemia. An optimized estimate of iCa from tCa with non-esterified fatty acids (NEFA), beta-hydroxybutyric acid, cholesterol, and phosphorous as co-predictors is still poorly satisfying.

## 1. Introduction

The synthesis and secretion of colostrum by dairy cows in the first days after calving leads to a large demand for Ca^2+^ in the first days postpartum [1]. Several homeostatic mechanisms need to adapt in this period to maintain the Ca^2+^ plasma pool in the light of Ca^2+^ requirements for colostrum and milk [2]. These mechanisms need to be effective because calcium plays a critical role in many physiological processes, including immune status, blood clotting, nerve impulse transmission, and muscle contraction [3]. However, about 5 to 10% of all multiparous cows show clinical signs of hypocalcemia or milk fever in the period after calving, and another 30 to 60% suffer from subclinical hypocalcemia with total serum calcium levels below 2 mmol·L^−1^ [4,5]. If the mammary need for blood calcium is not compensated quickly and adequately, hypocalcemia occurs and increases the risk for metritis, displaced abomasum, and other secondary diseases [4].

In blood plasma, calcium exists in three different fractions: about 50% is present in the free ionized form; 40% is protein-bound; and 10% is complexed with anions such as lactate, citrate, inorganic phosphate, and bicarbonate. These three fractions are in balance with each other [6], but only the ionized form is available for the maintenance of calcium homeostasis [7].

In the absence of clinical signs of milk fever, the measurement of blood Ca^2+^ is the key tool to recognize subclinical hypocalcemia [8,9]. Since only the ionized calcium (iCa) is biologically active, the concentration of iCa is more relevant than the concentration of total calcium (tCa) [10]. The determination of plasma iCa concentrations is well established for patient care in human medicine [6]; however, total calcium is usually measured in veterinary medicine [2]. The latter is technically less challenging because tCa can be measured hours after sampling or after freezing serum samples. By contrast, iCa must be measured immediately from gas-tight containers since the proportion of protein-bound calcium decreases with increasing pH, implying that changes in blood gases affect the concentration of iCa [11,12].

Some studies investigated the relationship between ionized calcium and total calcium in calves [13,14] or in dogs [15]. For adult dairy cows, previous studies provided the first indication that the relationship between tCa and iCa may change at the time after calving, so that ionized calcium accounts for a larger proportion of Ca^2+^ postpartum [3,16,17]. However, all those previous studies included cases of clinical milk fever together with measures to prevent or treat hypocalcaemia. In the present study, we tested the hypothesis that a discrepancy between tCa and iCa is evident even in apparently healthy multiparous dairy cows postpartum that received no hypocalcemia prophylaxis from either anionic salts or low-calcium diet prepartum.

## 2. Materials and Methods

Repeated blood samples around calving were taken from 14 Holstein Friesian cows on a dairy farm in Bavaria, Germany with an average daily milk yield of 37.5 kg/day over the first 14 d of lactation. Five cows were in their second lactation and nine in their third lactation. The farm had not included a management strategy against hypocalcemia like anionic salts or low calcium diet antepartum. Neither calcium infusions nor oral calcium supplementation was used in the study cows. Exclusion criteria included fever, paresis, reduced feed intake, poor condition, mastitis, and metritis.

The composition of the partial mixed ration (PMR) is shown in Table 1. After calving, cows additionally received two different types of concentrate feed in the automatic milking system. The amount of concentrates increased linearly in the first 15 days after calving from 2 kg/d to 4 kg/d for concentrate A and from 0.1 kg/d to 0.3 kg/d for concentrate B. The composition of concentrate feed is also given in Table 1.

Blood was taken at −2 (±1), 0 (+1), +2 (±1), +4 (±1), +7 (±1), and +14 (±1) days relative to calving. Day 0 was always the day of calving or, in a few exceptions, the day after calving if cows calved at night. Two consecutive blood samples were drawn from a coccygeal vessel using standard venipuncture without anesthesia into one 9-mL vacutainer with a separator for serum and another 9-mL lithium-heparin vacutainer for whole blood samples (Greiner Bio-One International GmbH, Kremsmünster, Austria). To ensure the most anaerobic collection possible, the vacutainer was first removed from the needle and then the needle was removed from the vessel to avoid aspiration of air [18].

Ionized calcium was measured from the heparinized blood sample using an ion-selective electrode (Stat Profile Prime blood gas analyzer, NOVA Biomedical GmbH, Mörfelden-Walldorf, Germany). The first measurement of iCa was taken directly from the anaerobic heparinized vacutainer (iCa_H1) followed by a second measurement from a sodium heparin-coated capillary (Hirschmann Laborgeräte GmbH and Co. KG, Eberstadt, Germany) filled with blood from the same vacutainer to achieve double heparinization (iCa_H2). The latter procedure was intended to test the impact of the heparinization protocol on the concentration of ionized calcium. Repeatability between subsequent measurements of iCa from the vacutainer had a coefficient of variation (CV) of 0.61%. Furthermore, the Stat Profile Prime blood gas analyzer simultaneously measured pH, pCO_2_, pO_2_, hematocrit, sodium, potassium, chloride, glucose, and lactate. The Stat Profile Prime additionally calculated the normalized calcium (nCa) for a hypothetical pH of 7.4 using the following formula: log [iCa]_7.4_ = log [Ca^2+^]_x_ − 0.24 (7.4 − x), where x is the measured pH of the sample, [Ca^2+^]_X_ is the concentration of ionized calcium in the sample at the measured pH, and [iCa]_7.4_ is the normalized concentration of ionized calcium at pH 7.4.

After clotting, blood was centrifuged at 1500× *g* for 10 min (Centrifuge EBA 270, Andreas Hettich GmbH and Co.KG, Tuttlingen, Germany) and serum aliquots were frozen at −20 °C until further analysis. In serum samples, concentrations of total calcium were determined photometrically at a wavelength of 660 nm [19] using an automatic biochemistry analyzer (Indiko, Thermo Fisher Scientific, Waltham, MA USA). The following serum parameters were also analyzed by the Indiko system using different photometric methods: phosphorus [20], magnesium [21], non-esterified fatty acids (NEFA) [22], β-hydroxybutyric acid (BHB) [23], aspartate aminotransferase [24], glutamate dehydrogenase [25], alkaline phosphatase [26], bilirubin [27], cholesterol [28], blood urea nitrogen [29], creatinine [30], and creatine kinase [24]. The repeatability for total calcium had a CV of 1.37%. Total protein and albumin concentrations were measured from serum via spectral analysis (Catalyst One, IDEXX GmbH, Ludwigsburg, Germany). Albumin and total protein were determined at wavelengths of 650 and 560 nm, respectively.

To describe the changes of calcium over time relative to baseline, individual values from 2 d before calving were set to 100% for each cow and relative values from other days were calculated as percent of baseline.

Statistical analyses were performed using the software SigmaPlot 11.0 (Systat Software GmbH, Erkrath, Germany). Correlation between calcium measurements from different methods were estimated using linear correlation analyses. Time-dependent changes in absolute or relative calcium concentrations around parturition were analyzed by two-way repeated measures (RM) ANOVA followed by Student-Newman-Keuls’ post-hoc test. One-way repeated measures ANOVA and Student-Newman-Keuls’ test were used to analyze changes in the ratio between ionized and total calcium around parturition. Data of the blood minerals, biochemicals, and enzymes were tested for normality (Shapiro–Wilk test) and equal variance. On passing both tests, data were compared using RM ANOVA; if one of these tests failed, Friedman’s RM ANOVA on ranks was used. Tukey test was used for multiple post-hoc comparison.

A multivariable prediction model for iCa was calculated using the generalized linear mixed effect models (SPSS 26, SPSS Inc., Chicago, IL, USA). All serum and whole blood parameters were included in the first model with subsequent stepwise elimination of non-significant parameters. Only significant parameters were kept, and models were compared using the Akaike information criterion. As the first model did not produce satisfactory results, a second multivariable regression model was tested between all serum and whole blood parameters and the ratio of iCa_H1:tCa to examine the influence of different parameters on the proportion of the free form of calcium in total calcium. Using backward elimination, non-significant parameters were excluded from the model as described for the first model.

## 3. Results

None of the animals included in the study showed clinical signs of illness; in particular, no signs of hypocalcemia, such as ataxia or paresis, reduced feed intake, tachycardia, or cold extremities were observed. Cows with incomplete data were excluded from the data set, i.e., only cows with complete data sets for each time point were included in the analyses. The supporting parameters analyzed from serum or whole blood (pH, pCO_2_, pO_2_, hematocrit, sodium, potassium, chloride, glucose, lactate, phosphorus, magnesium, NEFA, BHB, aspartate aminotransferase, glutamate dehydrogenase, alkaline phosphatase, bilirubin, cholesterol, urea, creatinine, and creatine kinase; Table 2) did not indicate disease, except for five cows that had BHB > 1.2 mmol·L^−1^ on single days, indicating mild subclinical ketosis.

**Figure 1:**
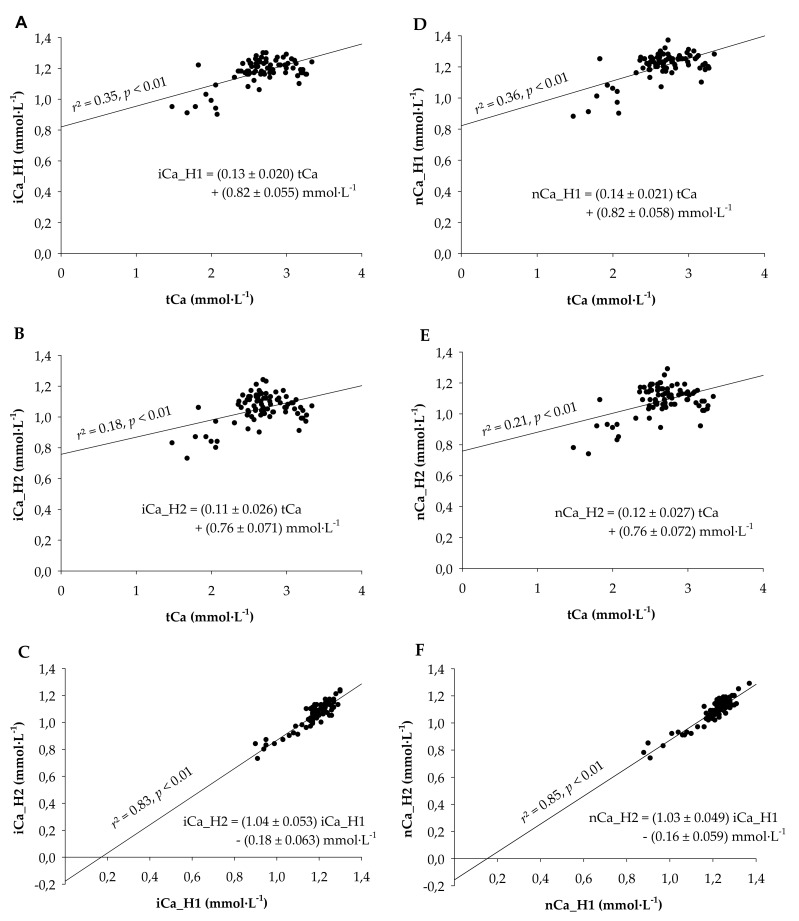
Correlations and regression analyses (**A**) between plasma ionized calcium measured from a heparinized vacutainer (iCa_H1) and serum total calcium (tCa), (**B**) between plasma ionized calcium measured from a heparinized capillary filled from a heparinized vacutainer (iCa_H2; double heparinization) and serum total calcium (tCa), and (**C**) between iCa_H2 and iCa_H1. (**D**–**F**) Regression analyses corresponding to graphs A-C where ionized calcium values were replaced by the ionized calcium values normalized to a pH of 7.4. All slopes and intercepts were different from zero (*p* < 0.01). Blood was taken from 14 multiparous Holstein-Frisian cows between −2 and 14 days relative to calving.

The mean values for tCa, iCa_H1, and iCa_H2 were 2.66 ± 0.041, 1.18 ± 0.009, and 1.05 ± 0.011, respectively. When plotting all values from all measurement days, correlations were identified for iCa_H1 and tCa (*r*^2^ = 0.35; *p* < 0.01), iCa_H2 and tCa (*r*^2^ = 0.18; *p* < 0.01), and iCa_H2 and iCa_H1 (*r*^2^ = 0.83; *p* < 0.01). Linear regressions between ionized and total calcium concentrations were estimated as iCa_H1 = (0.13 ± 0.020) tCa + (0.82 ± 0.055) mmol·L^−1^ and iCa_H2 = (0.11 ± 0.026) tCa + (0.76 ± 0.071) mmol·L^−1^, with all slopes and intercepts being different from zero (*p* < 0.01; Figure 1A,B). Although the slopes and intercepts of the latter two regressions were not different from each other (*p* > 0.1), the regression between iCa_H2 and iCa_H1 (iCa_H2 = (1.045 ± 0.053) iCa_H1 − (0.18 ± 0.063) mmol·L^−1^) had an intercept different form zero (*p* < 0.01), indicating that iCa_H2 was systematically underestimating iCa_H1 (Figure 1C). We also determined the linear regression between the different types of measurements with the pH-corrected (i.e., normalized) values of ionized calcium (nCa). Since no clear difference was visible (Figure 1D–F), we decided to use the uncorrected ionized calcium for all further analyses.

Because the high intercept and the low *r*^2^-value for the prediction of iCa_H1 from tCa were not satisfying, a multivariable regression using all further minerals, biochemicals, and enzyme activities was performed with the intention to improve the prediction model. After excluding all non-significant variables, the following equation remained: iCa_H1 = (0.16 ± 0.018) tCa mmol·L^−1^ − (0.004 ± 0.0022) Cl^-^ mmol·L^−1^ − (0.28 ± 0.135) pH + (3.3 ± 1.05) mmol·L^−1^ (*r*^2^ = 0.40, *p* < 0.001). The similar slopes for tCa (0.16 vs. 0.13) and the only slightly improved *r*^2^ (0.40 vs. 0.35) indicated that predicting iCa_H1 with this more complex equation was only gradually superior to predicting iCa_H1 from tCa alone.

Proceeding from the assumption that other blood biochemicals may complex Ca and thereby affect the ratio of iCa:tCa rather than the absolute values of iCa, we subsequently tested the multivariable regression between the iCa_H1:tCa ratio and all measured blood parameters. After stepwise elimination of non-significant parameters, the ratio of iCa_H1:tCa could be estimated as iCa_H1:tCa = −(0.058 ± 0.022)·|NEFA (mmol·L^−1^)| − (0.029 ± 0.017) |BHB (mmol·L^−1^)| − (0.039 ± 0.008) |cholesterol (mmol·L^−1^)| − (0.025 ± 0.009) |phosphorous (mmol·L^−1^)| + (0.0065 ± 0.0013) |albumin (g/L)| + (0.442 ± 0.045) (*r*^2^ = 0.58, *p* < 0.001). Because the positive correlation between iCa_H1:tCa and albumin was not logical, the multivariable correlation was also estimated without consideration of albumin. This resulted in a model with iCa_H1:tCa = −(0.071 ± 0.025)⋅ |NEFA (mmol·L^−1^)| − (0.045 ± 0.019) |BHB mmol·L^−1^)| − (0.033 ± 0.001) |cholesterol (mmol·L^−1^)| − (0.025 ± 0.001) |phosphorous (mmol·L^−1^)| + (0.637 ± 0.026) (*r*^2^ = 0.43, *p* < 0.001).

To understand the timeline of changes around parturition, mean values for total and ionized calcium were analyzed for each sampling day (Table 3). Two-way RM ANOVA revealed effects of time (*p* < 0.01) and measurement method (*p* < 0.01) with two-way interaction of time × measurement method (*p* < 0.01). The interaction was based on generally higher values for tCa > iCa_H1 at all time points and a decrease in calcium concentration at the day of calving, the latter being significant for only tCa (*p* < 0.05). When calculating the ratio iCa_H1:tCa, the mean ratio for iCa_H1:tCa was 0.45 ± 0.002; however, the proportion of ionized calcium in total calcium increased sharply at the day of calving (*p* < 0.001) and returned to basal values within 7 d (Table 3).

**Figure 2:**
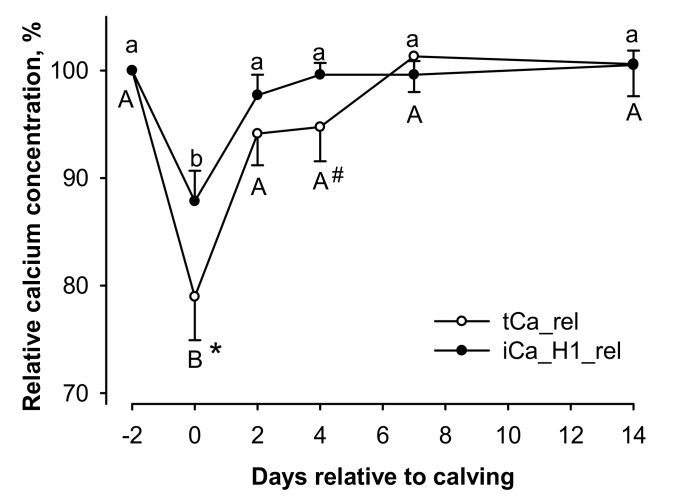
Influence of days in milk on total serum calcium (tCa_rel) and ionized calcium measured from heparinized vacutainer (iCa_H1_rel) relative to the prepartum values at day −2 (100%). Relative values were affected by time (*p* < 0.01) with two-way interaction of time × measurement method (*p* < 0.05). A,B different capital letters and a,b small letters indicate differences for tCa_rel and iCa_H1_rel, respectively (*p* < 0.05, each). #,* hash key and asterisk indicate a trend for difference (*p* < 0.1) and difference to the corresponding value of iCa_H1_rel (*p* < 0.05), respectively.

To demonstrate the discrepancy between the time-dependent changes in tCa and iCa_H1 around calving even clearer, the relative changes in tCa (tCa_rel) and iCa_H1 (iCa_H1_rel) were plotted with day −2 before calving as reference (Figure 2). In this model, relative calcium levels were affected by time (*p* < 0.01) with two-way interaction of time × measurement method (*p* < 0.05). The interaction was based on decreases in calcium concentration from day −2 before calving to the day of calving for each measurement method (*p* < 0.01) with recovery to basal values thereafter. However, tCa_rel showed lower relative calcium values than iCa_H1_rel at the day of calving (*p* < 0.05), which was still visible as a trend at day 4 after calving (*p* = 0.061; Figure 2).

## 4. Discussion

A deeper understanding of the calcium dynamics around calving is essential to develop effective prophylactic and therapeutic strategies for hypocalcemia of dairy cows. It has been proposed that iCa provides a more useful and relevant estimate of the calcium gap at calving and helps prevent incorrect prophylactic calcium infusions in treatment regimens of hypocalcemia [31]. This is highly plausible, although clinical benefits and better predictability of disease incidences by diagnostic use of iCa compared to tCa have yet to be proven. To assess the necessity of such research, the present study analyzed the relationship between iCa and tCa, which provides the rationale behind any diagnostic use of those calcium measurements from blood. We also addressed the effect of heparinization on iCa that is rarely considered. Our intention was to describe those effects and relationships without any external bias; hence, the study was performed on dairy cows that were not subject to any hypocalcemia prevention strategy, oral calcium supplementations, or intravenous calcium infusions.

The value of a precise description of the animal’s calcium status around calving is evident. Hypocalcemia implies economic losses, primarily due to secondary diseases and decreased fertility [1,32]. Furthermore, cows suffering from subclinical hypocalcemia require longer time for calf delivery with associated risks for calf health and vitality [33]. Reliable detection of subclinical hypocalcemia could improve prophylactic strategies and increase the willingness to introduce them. This could finally lower economic losses and improve herd health status.

The measurement of tCa is routinely used in the diagnosis of suspected subclinical hypocalcemia in dairy cows [2,34]. Only very few studies used iCa [4,35,36], although iCa is more relevant because it represents the biologically available form of calcium. The present study clearly proves that one cannot simply extrapolate iCa from tCa in the period around calving. The linear correlation model between iCa and tCa did not show any satisfying precision, which is in line with studies from human medicine [6]. The use of normalized calcium did not improve the predictive power. The latter is coherent with a similar study by Yogeshpriya et al. where the uncorrected iCa was identified as a more valid and precise marker for calcium homeostasis than nCa [37]. Normalized calcium was thus not considered any further in the present study.

The unsatisfactory precision when predicting iCa from tCa alone originated from different degrees of decrease in tCa and iCa at the day of calving. Previous studies mentioned similar effects and suggested other factors influencing the proportion between iCa and tCa [17,38]. Therefore, we further analyzed a great portfolio of other ions, biochemicals, enzymes, gases, and pH in blood to perform a multivariable correlation analysis. However, the only two other factors that showed significant relationship to iCa (except for tCa) were plasma chloride concentration and pH. Unfortunately, the inclusion of these two variables into a multivariable regression model provided only slight improvement when estimating iCa from tCa, which makes their usability equally questionable as the simple linear model. Moreover, because pH always requires measurement from fresh blood with similar or identical equipment as used for iCa, there is not really an argument for improving the estimate of iCa from serum tCa by correction for pH. Instead of using fresh blood to analyze pH for indirect estimation of iCa, it would be more appropriate to use an anaerobically taken sample immediately for direct measurement of iCa [11,39].

As the lack of precision of the multivariable estimation of iCa from a portfolio of other blood values was rather disappointing, we hypothesized that other blood minerals and biochemicals may not impact on iCa directly but rather impact on the ratio of iCa:tCa. Therefore, we additionally calculated the multivariable regression between iCa:tCa and all measured blood parameters. This model identified several parameters with plausible influences on the amount of biologically available iCa. Among these were NEFA and BHB, which are anions that are able to bind calcium. Especially, long chain fatty acids can complex Ca into Ca soaps [40], which subsequently decreases the fraction of iCa within tCa. High values of NEFA and BHB are signs of a negative energy balance that increase the risk for secondary diseases like retained placenta [41,42] and milk fever [43]. A novel finding of the present study was that the increased incidence of milk fever after parturition may be partly caused by complexing of plasma calcium by the high levels of BHB and NEFA as suggested by the negative correlation of iCa:tCa with NEFA and BHB.

We further identified negative correlation between iCa:tCa and serum phosphorous. The influence of phosphorus at the level of ionized calcium became visible immediately after calving in the present study. Serum phosphorus concentration dropped sharply, whereas the proportion of ionized calcium showed a significant increase. This is a very interesting finding considering that decreases in serum concentrations of tCa and phosphorus often concur after parturition and are also typical for experimentally induced hypocalcemia [44,45]. The present study suggests that, despite being unfavorable for the cow as such, the decrease in serum phosphorus concentration after parturition helps ameliorating the effects of hypocalcemia due to increases in the fraction of iCa within tCa.

The negative relationship observed between iCa:tCa and serum cholesterol has less obvious explanations. Nonetheless, it is known that cholesterol often co-localizes with Ca-trapping mechanisms, e.g., in human atherosclerosis [46] which makes the negative relationship between iCa:tCa and serum cholesterol, at least, functionally plausible. Serum cholesterol concentration shows great changes around parturition [47] and was proposed to be a good predictor of energy balance status during early lactation [48].

Finally, the positive relationship between the amount of ionized calcium and albumin was unexpected and the only implausible relationship in this model. Almost 50% of calcium is bound to protein, especially albumin [6]. Therefore, higher serum protein levels should decrease the proportion of iCa within tCa, meaning that a negative correlation would have been expected between iCa:tCa and serum albumin. One possible explanation for the observed positive correlation may be that the correlation between ionized calcium and albumin is known to vary greatly between individual cows [49].

Proceeding from the assumption that the observed positive correlation between iCa:tCa and serum albumin was based on artifact, we tested how the multivariable model for iCa:tCa estimation would perform without serum albumin. Unfortunately, the omission of albumin decreased the precision of prediction to *r*^2^ = 0.43, which was worse than with inclusion of albumin but better than all other models tested. From this, it may be deduced that iCa may be estimated as iCa = (0.637 − 0.0714·|NEFA (mmol·L^−1^)| − 0.0452 |BHB (mmol·L^−1^)| − 0.0332 |cholesterol (mmol·L^−1^)| − 0.0253 |phosphorous (mmol·L^−1^)|) tCa if direct measurement of iCa is not possible under field conditions. However, as the accuracy of the above estimate is still very limited, it needs to be concluded that there is currently no reliable substitute for direct measurement of iCa. Direct measurement of iCa is more demanding because a fresh whole blood sample needs to be sampled in an air-tight container to maintain blood pH and special, preferably on-site equipment with ion-sensitive electrodes is required. Nonetheless, the present study demonstrates that further research needs to take that effort to explore the expectable benefits of iCa for hypocalcemia diagnosis.

A final point for consideration is that blood preservation affects iCa. Whole blood samples are usually collected into containers with anticoagulants like EDTA or heparin [50,51,52]. EDTA, citrate, and oxalate should be avoided as they effectively chelate calcium [50]. Thus heparin is the anticoagulant of choice for samples dedicated for iCa measurements. However, heparin also binds small amounts of calcium, which is usually not appreciated in routine analysis [53,54]. To demonstrate the partial calcium chelation by heparin [55], we used a double heparinization approach with conventionally available consumables for such measurements. This approach showed a very high correlation of simple and double-heparinization results; however, a significant intercept of −0.18 mmol·L^−1^ largely corresponded to the difference in the mean values of iCa_H1 and iCa-H2 (0.13 mmol·L^−1^). It indicated that the second heparinization step systematically reduced iCa by ~0.13 mmol·L^−1^, which is ~11% of the mean iCa. In order to define reference ranges and to reliably evaluate results, it is thus necessary to use blood sampling methods with standardized heparinization.

## 5. Conclusions

Hypocalcemia is a widespread disease in postpartum dairy cows. The present study demonstrates that the proportion of iCa within tCa varies greatly in the critical period around parturition, thus hindering precise estimation of iCa from tCa. Although we demonstrate here that the estimate of iCa from tCa can be improved by implementing NEFA, BHB, cholesterol, and phosphorous in the prediction formula, the precision of the estimate is still far from satisfactory. This implies that direct measurement of iCa cannot really be replaced by other approaches. Therefore, we recommend to thoroughly assess the possible benefits of using iCa in future diagnosis of postpartum hypocalcaemia. Since iCa reflects the biologically available form, it is expected that iCa would be a better predictor of postpartum disease probability than tCa. An extended database on iCa dynamics around calving is desirable for a better understanding of hypocalcemia and milk fever, including their prevention and treatment.

## Figures and Tables

**Table 1:** Diet ingredients and chemical composition of partial mixed ration (PMR) fed to dairy cows antepartum and postpartum, as well as of concentrates supplied by the automatic milking system postpartum.

Feed Items (%)	PMR Antepartum	PMR Postpartum	Concentrate A	Concentrate B
Hay	9.9	6.2	0	0
Maize silage	23.4	24.5	0	0
Grass silage	60.4	63.0	0	0
Maize	1.5	1.5	35	33
Wheat	0	0	10	16
Barley	1.5	1.5	0	14.5
Rapeseed meal	1.5	1.5	20	22.5
Soya extraction meal	0	0	9	10
Maize gluten meal	0	0	7	0
Dried molasses	1.3	1.3	6	0
Malt sprouts	0	0	5	0
Biscuit flour	0	0	3	0
Sugar beet vinasse	0	0	2	2.6
Sugar beet pulp	0.2	0.2	1	0
Mineral mix	0.3 ^1^	0.3 ^1^	0.1 ^2^	0.1 ^2^
Calcium carbonate	0	0	1.5	0.9
Sodium carbonate	0	0	0	0.4
Sodium chloride	0	0	0.4	0
Chemical composition (g/kg dry matter) ^3^				
Dry matter	417	384	901	899
Crude protein	135	149	215	218
Crude fat	30	34	45	46
Starch	137	145	338	315
NDF	448	406	247	265
ADF	270	247	108	112
ADL	27	24	22	24
Calcium	5.7	6.1	8	10
Magnesium	2.3	2.5	7	8
Phosphorous	3.4	3.9	3	3
DCAD (meq/kg)	+253	+285	+60	+88

^1^ The mineral mix contained per kg: 44 g calcium carbonate; 19 g sodium chloride; 16 g calcium sodium phosphate; 12 g magnesium oxide; 3 g magnesium sulphate; 400,000 I.U. vitamin A; 65,000 I.U. vitamin D3; 4000 mg vitamin E; 250 mg vitamin C; 80 mg vitamin B1; 40 mg vitamin B2; 20 mg vitamin B6; 200 μg vitamin B12; 5000 mg niacinamide; 80 mg calcium D-pantothenate; 16,600 μg biotin; 16,500 mg choline chloride; 5000 mg zinc, of which 4500 mg was glycine zinc chelate hydrate (solid) and 500 mg was zinc oxide; 5000 mg manganese, of which 1500 mg was glycine-manganese chelate hydrate and 3500 mg was manganese(II) oxide; 1000 mg copper, 750 mg of which was copper(II) glycine chelate hydrate (solid) and 250 mg of which was copper(II) sulphate pentahydrate; 150 mg iodine as calcium iodate, anhydrous, and 15 mg cobalt as coated cobalt(II) carbonate granules; 25 mg selenium, 3 mg of which was selenium yeast from *Saccharomyces cerevisiae* NCYC R397, inactivated, and 22 mg of which was sodium selenite. ^2^ The mineral mix contained per kg: 9000. I.U. vitamin A; 1000 I.U. vitamin D3; 10 mg vitamin E; 13 mg copper(II) sulphate; pentahydrate; 0.75 mg iodine as calcium iodate, anhydrous; 0.15 mg cobalt as coated cobalt(II) carbonate granules; 30 mg manganese as manganese(II) oxide; 45 mg zinc as zinc oxide; and 0.12 mg selenium as sodium selenite. ^3^ Chemical composition was analyzed in samples of PMR collected at monthly intervals, and each concentrate was collected three times in the experimental period.

**Table 2:** Non-esterified fatty acids (NEFA), β-hydroxybutyric acid (BHB), aspartate aminotransferase (AST), glutamate dehydrogenase (GLDH), alkaline phosphatase (ALKP), bilirubin (TBIL), cholesterol (Chol), blood urea nitrogen (BUN), creatinine (Crea), creatine kinase (CK), phosphorus (P), magnesium (Mg), total protein (TP), and albumin (Alb) of cows in the period around calving.

**Day**	**NEFA**	**BHB**	**AST**	**GLDH**	**ALKP**	**TBIL**	**Chol**
	**µmol·L^−1^**	**µmol·L^−1^**	**U·L^−1^**	**U·L^−1^**	**U·L^−1^**	**µmol·L^−1^**	**mmol·L^−1^**
−2	290 ^b^	686	79 ^c^	10.1 ^b,c^	51.9 ^a,b^	2.84 ^b^	2.02 ^b,c^
0	490 ^a^	757	95 ^b^	10.7 ^c^	53.4 ^a^	5.21 ^a^	1.70 ^c^
2	387 ^a,b^	800	108 ^a,b^	13.2 ^a,b,c^	50.6 ^a,b^	3.84 ^a,b^	1.81 ^c^
4	479 ^a,b^	831	111 ^a^	17.2 ^a,b^	48.1 ^a,b^	3.84 ^a,b^	1.99 ^b,c^
7	448 ^a,b^	888	112 ^a^	22.2 ^a^	44.4 ^b^	3.69 ^a,b^	2.44 ^a,b^
14	411 ^a,b^	923	101 ^a,b^	23.9 ^a,b^	42.7 ^b^	3.15 ^b^	2.97 ^a^
SEM	22.5	31.8	2.6	1.23	1.2	0.177	0.037
*p*-Value	0.034	0.053	<0.001	<0.001	0.002	0.003	<0.001
**Day**	**BUN,**	**Crea,**	**CK,**	**P**	**Mg**	**TP**	**Alb**
	**mmol·L^−1^**	**µmol·L^−1^**	**U·L^−1^**	**mmol·L^−1^**	**mmol·L^−1^**	**g·L^−1^**	**g·L^−1^**
−2	3.9	101 ^a^	146	2.16 ^a^	0.98 ^a,b^	73 ^b^	28 ^b^
0	3.58	102 ^a^	197	1.42 ^b^	1.10 ^a^	74 ^b^	29 ^a,b^
2	3.5	91 ^a,b^	194	2.02 ^a^	0.95 ^b^	73 ^b^	28 ^a,b^
4	3.96	87 ^b^	154	2.10 ^a^	0.92 ^b^	76 ^b^	29 ^a,b^
7	3.89	95 ^a,b^	138	2.10 ^a^	0.97 ^a,b^	76 ^a,b^	29 ^a,b^
14	3.72	78 ^b^	150	2.08 ^a^	1.05 ^a,b^	79 ^a^	30 ^a^
SEM	0.098	1.4	12.6	0.05	0.016	0.6	0.4
*p*-Value	0.9	<0.001	0.11	<0.001	0.001	<0.001	0.028

Values are means and pooled SEM. a–c values within a row with different superscripts differ significantly at *p* < 0.05.

**Table 3:** Time- and method-dependent changes in ionized calcium measured from heparinized vacutainer (iCa_H1), total serum calcium (tCa) and the resulting ratio iCa_H1:tCa.

Sampling Day	iCa_H1 (mmol·L^−1^)	tCa (mmol·L^−1^)	Ratio iCa_H1:tCa
d−2	1.21	2.83 ^a^	0.43 ^b^
d0	1.07	2.26 ^b^	0.48 ^a^
d2	1.18	2.65 ^a^	0.45 ^ab^
d4	1.21	2.66 ^a^	0.46 ^ab^
d7	1.21	2.86 ^a^	0.43 ^b^
d14	1.21	2.83 ^a^	0.43 ^b^
SEM	0.009	0.041	0.006

Values are least square means (LSM) and pooled standard error of mean (SEM). iCa_H1 = ionized calcium measured from vacutainer, tCa = total serum calcium. Data for iCa_H1 and tCa were affected by time, measurement method, and their two-way interaction (*p* < 0.01, each). The ratio of iCa_H1:tCa was affected by time (*p* < 0.01). a–b different superscripts within one column indicate differences between sampling days at *p* < 0.05. Values for tCa were higher than values for iCa_H1 at all times.

## Data Availability

Data is contained within the article.
